# Editorial: Nutrition in the Regulation of Muscle Development and Repair

**DOI:** 10.3389/fphys.2022.853007

**Published:** 2022-02-24

**Authors:** Olasunkanmi A. J. Adegoke, Yan Huang, Xing Fu, Stephen Mora

**Affiliations:** ^1^School of Kinesiology and Health Science and Muscle Health Research Centre, York University, Toronto, ON, Canada; ^2^Department of Animal Science, Division of Agriculture, University of Arkansas, Fayetteville, AR, United States; ^3^School of Animal Sciences, Louisiana State University Agricultural Center, Baton Rouge, LA, United States

**Keywords:** skeletal muscle, repair, regeneration, amino acids, fatty acids, micronutrients, satellite cells, insulin resistance

The significance of skeletal muscle on whole body metabolism and wellbeing is underlined by the fact that when muscle mass, quality and/or functions are suboptimal, there are implications for whole body wellbeing. It is unequivocal that specific nutrients can regulate the growth of skeletal muscle, either alone or in combination with resistance exercise. Mechanisms regulating formation of muscle cells, either during development or muscle repair/regeneration postnatally, are also well understood. However, although formation of new post-mitotic muscle cells (myotubes) requires synthesis of new proteins, and that amino acids (AA) are required for protein synthesis, not much is understood about the link between nutrients (micro and macro) and formation of new muscle cells. Related to this is the contribution of amino acid metabolism to muscle and whole-body well-being. From food production and nutrition perspectives, it is critical that foods be not only nutritious but be desirable: of what value is a highly nutritious/healthy food if consumers find it unsavory? This Research Topic is a collection of articles on these issues.

Two reviews in this collection focus on the role of nutrients in regulating muscle satellite cells and muscle regeneration. In one, Blum et al. reviewed evidence and gaps regarding nutrient and metabolic determinants of muscle regeneration. The nonessential amino acids serine and glycine, as well as vitamins A and D, are candidate nutrients. The review also discussed the impact of whole foods (blueberry supplements) and obesity on satellite cell integrity. In the second review, Latham et al. discussed evidence for the functions of vitamin D in skeletal muscle regeneration following injury. Because vitamin D can regulate maintenance of satellite cell (SC) integrity, it is tempting to speculate a role for vitamin D in the preservation of muscle regenerative ability, especially during aging. Studies on the effects of nutrients on muscle anabolism have typically focused on macronutrients. However, macronutrient and energy metabolism depends on micronutrients (especially the B vitamins) status because of the roles of the latter as cofactors. He et al. examined the effects of maternal methyl donor mix (MET, a mix of folic acid, methionine, choline, vitamins B6 and B12) supplementation in pregnant pigs. Piglets from dams that consumed the supplement had increased body weight and myofiber diameter, though with reduced fiber number. These improvements occurred in parallel with increased circulating levels of relevant hormones and signaling proteins.

Two reports documented the effects of betaine (trimethylglycine), a feed supplement that is used in livestock production, on growth, and meat/carcass yield and quality in piglets. Betaine functions primarily as an osmolyte and methyl donor (Stuart and Craig, 2004). In one report, the authors showed that betaine increased piglet feed intake (consistent with increased blood gastrin level) and growth, though feed/gain ratio was not different. There were varied and modest effects on intramuscular lipid (IMF) content, but no effects on muscle ω3 PUFA levels. Given the significance of ω3 fatty acids in human health, identifying livestock management practices that do not only enhance flavors but also promote the incorporation of “good” lipids are desirable. In the 2nd study, betaine supplementation increased lean weight and measures of meat quality. It also altered muscle fiber type. The data are consistent with the supplement having a positive effect on meat production and quality. Because increased blood levels of alanine and aspartic aminotransferases were observed, it will be useful to examine what the effect of the supplement will be on animal health. In a related study, Wang et al. used meta-analysis to review the effect of polyunsaturated fatty acids (PUFA) on meat quality of pigs. Evidence suggests that PUFA, specifical conjugated linoleic acid (CLA, a derivative of linoleic acid, an ω6 fatty acid,) and linseed (a type of flax seed from which α-linolenic acid, an ω3 FA, is derived) can improve IMF (a main measure of meat quality) but not growth indices. Thus, more still needs to be done to identify practices that will improve both meat production and quality.

AAs are critical for the synthesis of proteins and compounds that are vital for muscle functions (e.g., creatine, carnitine, glutathione). Factors that regulate AA availability include transport, disposal into proteins and other compounds, oxidation, and their release from proteolysis. Acute studies showed a positive correlation between the expression of AA transporters and muscle protein synthesis (Drummond et al., 2011; Roberson et al., 2018). Roberson et al. examined whether chronic resistance exercise and leucine/whey protein concentrate would regulate protein synthesis and expression of amino acid transporters. Resistance exercise suppressed protein abundance of ATF4 (a transcription factor that normally induces the expression of AA transporters) but increased the expression of AA transporters LAT1 and PAT1. For LAT1, the effect of exercise was reduced in the two supplement groups. Surprisingly, C2C12 that overexpressed LAT 1 had reduced protein synthesis. It is also not clear why the overexpression of the transporter would lead to a reduction in BCKDHa, the enzyme that catalyzes the first irreversible step in branched-chain AA (BCAA) catabolism.

Exercise may modify gut microbiota, leading to the emergence of bacteria genera that favor adaption and improved exercise performance (Scheiman et al., 2019). Because the use of supplements is prevalent amongst athletes (Garthe and Maughan, 2018), Jaago et al. examined the effect of prebiotics mix on exercise-induced changes in microbiota. Prebiotic, but not training, reduced the diversity of some bacteria phyla by >95%. Although this was a study of *n* = 1, and there were no exercise-alone control groups, the data are hypothesis-generating regarding the impact of supplements on exercise and athlete health. It would also be interesting to examine what implications the changes in microbiota have on muscle health/functions. Given the effects of nutraceuticals on health, there is interest in examining their effects on muscle. Black chokeberry (aronia) is rich in phenolic compounds (Tolic et al., 2017; Shikov et al., 2021) and previous studies have documented its effect on health. Yun et al. showed that aronia fruit extracts increased MHC protein levels in C2C12 myotubes and reduced dexamethasone-induced atrophy. Mice treated with the extract had bigger and stronger muscles and better mitochondrial functions. Because animals treated with aronia had higher body weight, it would be interesting to know treatment effects on adipose tissue.

Muscle can regulate metabolism in other tissues through the release of muscle-derived myokines and metabolites (myometabolites) (Rai and Demontis, 2016). Hypoxanthine is a metabolite of purine and of ATP metabolism that is released during muscle contraction and is implicated in muscle fatigue. Yin et al. showed that the negative effects of hypoxanthine on fatigue was linked to reduced muscle glycogen and increased muscle lactate. These observations occurred along with increased expression of uncoupling protein 2 (UCP2) and reduced muscle ATP levels. When UCP2 was deleted in muscle, the effects of hypoxanthine were abated. Given the beneficial effects of exercise, and that increased levels of UCPs are generally perceived as desirable, how does one get the best of both worlds: benefits of exercise while limiting the negative effects of metabolites like hypoxanthine?

Impaired metabolism of BCAAs is linked to chronic diseases like insulin resistance, cardiovascular disease, and some cancers. Mann et al. reviewed the evidence and discussed emerging mechanisms of regulation of BCAA catabolism, including micro RNAs and posttranslational modifications of enzymes involved in BCAA catabolism. The review also discussed the impact of circadian rhythm on BCAA catabolism and the emerging observation that each of the BCAAs has unique metabolic imprints.

These articles shed more light on the roles that nutrients play in regulating muscle growth and regeneration, and how nutritional factors can regulate food (meat) production and quality (Figure 1). Some interesting hypotheses were generated, the testing of which will further advance the field.

**Figure 1:**
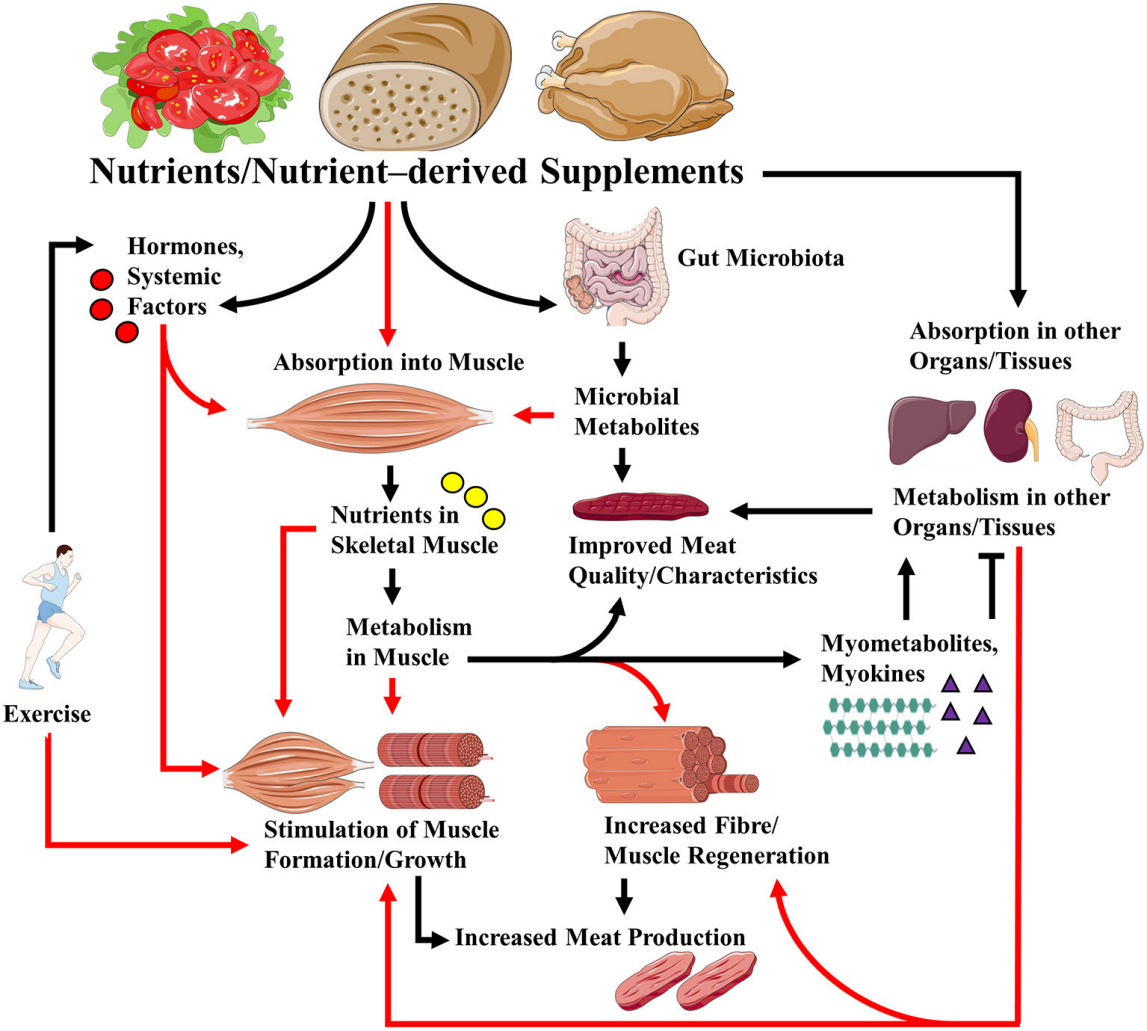
Links between nutrients/ nutrient-derived supplements and skeletal muscle growth/regeneration, health and meat quality. Nutrients and supplements derived from them can regulate muscle growth/regeneration, muscle metabolism, and, in the case of livestock production, meat quality *via* direct effects on muscle, and indirectly through their effects on hormones and systemic factors, their effects on other organs/tissues, and the gut microbiota. Metabolism of the nutrients/supplements in skeletal muscle can lead to the release of myokines and myometabolites that can affect (positively or negatively) metabolism in distal organs and tissues. Note that exercise may modulate the effects of nutrients and supplements *via* its actions on hormones, nutrient absorption, and gut microbiota. For simplicity, all the possible effects of exercise are not indicated. Red arrows indicate absorption into, and/or direct and indirect effects on muscle.

## Author Contributions

OAJA: conceptualization. OAJA and SM: writing and original draft preparation. OAJA, YH, XF, and SM: review and editing. All authors contributed to the article and approved the submitted version.

## Funding

This study was supported by a Natural Science and Engineering Discovery Grant (NSERC) to OAJA.

## Conflict of Interest

The authors declare that the research was conducted in the absence of any commercial or financial relationships that could be construed as a potential conflict of interest.

## Publisher's Note

All claims expressed in this article are solely those of the authors and do not necessarily represent those of their affiliated organizations, or those of the publisher, the editors and the reviewers. Any product that may be evaluated in this article, or claim that may be made by its manufacturer, is not guaranteed or endorsed by the publisher.
